# Modelling the spatial distribution of three *Portunidae* crabs in Haizhou Bay, China

**DOI:** 10.1371/journal.pone.0207457

**Published:** 2018-11-14

**Authors:** Jing Luan, Chongliang Zhang, Binduo Xu, Ying Xue, Yiping Ren

**Affiliations:** 1 College of fisheries, Ocean University of China, Qingdao, China; 2 Laboratory for Marine Fisheries Science and Food Production Processes, Pilot National Laboratory for Marine Science and Technology (Qingdao), Aoshanwei Town, Jimo, Qingdao, China; Fred Hutchinson Cancer Research Center, UNITED STATES

## Abstract

Crab species are economically and ecologically important in coastal ecosystems, and their spatial distributions are pivotal for conservation and fisheries management. This study was focused on modelling the spatial distributions of three *Portunidae* crabs (*Charybdis bimaculata*, *Charybdis japonica*, and *Portunus trituberculatus*) in Haizhou Bay, China. We applied three analytical approaches (Generalized additive model (GAM), random forest (RF), and artificial neural network (ANN)) to spring and fall bottom trawl survey data (2011, 2013–2016) to develop and compare species distribution models (SDMs). Model predictability was evaluated using cross-validation based on the observed species distribution. Results showed that sea bottom temperature (SBT), sea bottom salinity (SBS), and sediment type were the most important factors affecting crab distributions. The relative importance of candidate variables was not consistent among species, season, or model. In general, we found ANNs to have less stability than both RFs and GAMs. GAMs overall yielded the least complex response curve structure. *C*. *japonica* was more pronounced in southwestern portion of Haizhou Bay, and *C*. *bimaculata* tended to stay in offshore areas. *P*. *trituberculatus* was the least region-specific and exhibited substantial annual variations in abundance. The comparison of multiple SDMs was informative to understand species responses to environmental factors and predict species distributions. This study contributes to better understanding the environmental niches of crabs and demonstrates best practices for the application of SDMs for management and conservation planning.

## Introduction

Many fish populations have decreased in abundance and shifted distributions due to marine pollution, climate changes and over-exploitation [1–3]. In many marine ecosystems the declines of large predatory species have coincided with increase of small size, short-lived crustacean, including shrimps and crabs [4]. Moreover, the emerging economic values of crustacean species tend to be large and provide ample supports for local, small-scale fisheries [5–6]. For example, an increase of *Portunidae* contributed substantially to crab fisheries in the Yellow Sea over recent decades. Three crabs in the *Portunidae* family: *Charybdis bimaculata*, *Charybdis japonica*, and *Portunus trituberculatus*, are ecologically and economically valuable along the coastal area of China [7, 8]. Among them, *P*. *trituberculatus* has a larger body size, relative longer life span and are more migratory than *C*. *bimaculata* and *C*. *japonica* [9, 10]. Due to the functional natatorial legs of Portunids, “swimming crabs”, they have higher mobility than most other benthic crustaceans [11]. Consequent to their enhanced mobility, characterizing Portunid distribution is difficult modelling tasks. Unfortunately, despite their regional importance, there has been few studies to characterize the distribution and phenology of these species.

The spatial distributions of the crabs are influenced by environmental factors. For instance, temperature and salinity may influence overwintering of migratory crabs [12, 13], and in some special life history stage crabs might be more sensitive to salinity [7, 12]. Additionally, ranges of suitable temperature and substrate are strongly associated with their habitat preferences [14–16]. It has also been shown that dissolved oxygen influences the recruitment mortality of crabs, and therefore can serve as an important bottom-up driver of population dynamic [17]. Thereby, spatial distributional modelling studies that consider abiotic mechanisms are necessary to understand the environmental niches of crabs and assist in management and conservation planning.

Regarding spatial prediction, species distribution models (SDMs) are commonly used in ecology and biodiversity studies to predict species’ potential distribution [18]. For crabs, SDMs have been applied for estimating distributions of crabs living in different habitats (e.g. estuarine, intertidal zone and mangrove area), monitoring crab invasions [19, 20], forecasting fishing grounds [21, 22], reflecting modifications of typical habitats, identifying habitat suitability [12], and standardization of catch-per-unit-effort (CPUE) [23]. A wide range of statistical techniques are commonly used for SDMs, ranging from regression-based methods (linear regression, generalized additive models, and multivariate discriminant analysis [24, 25]), to non-parametric methods [26], such as machine learning (ML). It is established that although regression-based methods are straightforward to interpret, they are limited in their abilities to handle complicated relationships [24]. In many applications, ML can identify complex relationships flexibly and outperform regression-based methods in predictive capability [24]. In particular, previous ML studies on development of SDMs, using boosted regression trees (BRT), random forest (RF), maximum entropy (MaxEnt) and genetic algorithm, showed promise for predicting the native ranges of crabs [19, 20, 22] and many other marine species [22, 25, 27]. However, it should be noted that the models’ predictive performances depend not only on their algorithms [24], but also on study goals, spatial scales, sample sizes [28], distribution patterns [24, 29, 30], species characteristics [31], and the form of species responses to environmental changes [32]. Assessing the reliability of those models for various species is of great concern for their application. Therefore, a comparison of different MLs to traditional regression methods for multiple species is need to validate the practical application of SDMs.

In this study, we conducted a bottom-trawl crab survey and collected relevant environmental variables including temperature, salinity, depth, and sediment type. SDMs of three *Portunidae* crabs were developed using three modelling methods, including one traditional generalized additive model (GAM), and two ML approaches, random forest (RF) and artificial neural network (ANN) [33, 34, 35]. In order to understand these crabs’ spatial distributions and to assess the reliability of their distribution models, we identified the effects of environmental factors on three crabs and compared the performances among these models regarding fitting capability, predictability, and model stability. We paid special consideration to the effects of modelling method, species, and seasonality on model predictability. Finally, the distribution maps of three crabs were predicted using the developed models to support current regional fisheries management.

## Materials and methods

### Data collection

**Fig 1:**
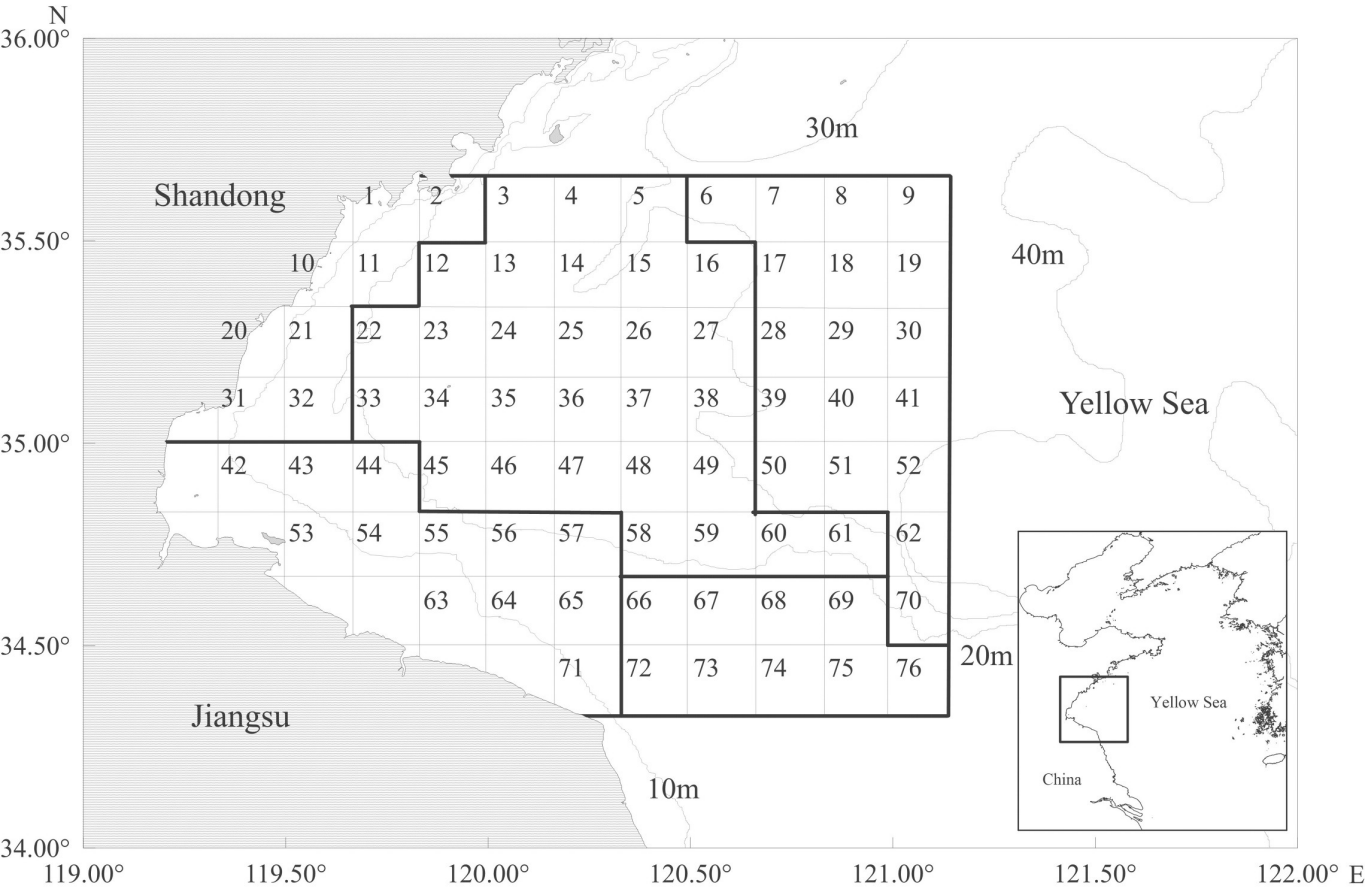
Map of survey stations for 2011, 2013–2016 in Haizhou Bay and adjacent waters.

The biomass data of the three crab species were collected in Haizhou Bay, China (34°25′−35°35′N, 119°25′−121°5′E), an open bay on the south-western Yellow Sea. (No specific permissions were required for the surveys, as the survey area was located in a typical fishing ground, in which there were no national parks or other protected area of wildlife. The surveys did not involve endangered or protected species). Haizhou Bay was a historically important fishing ground and served as a spawning and feeding habitat for many species in the 1980s [36]. Nonetheless, the ecosystem structure changed over past decades as a result of climate changes and increasing fishing pressure [37]. We conducted bottom trawl surveys in spring and fall of 2011, 2013, 2014, 2015 and 2016. A stratified random sampling design was used, in which the survey area was divided into five strata based on water depth (from 3.77m to 39.86m) and latitude (Fig 1). A total of 24 sampling sites in 2011 and 18 sites in the following years were chosen in each survey, covering the whole area of Haizhou Bay. We used otter trawl vessels of 162 kW and trawl nets with the cod-end mesh size of 17mm and width of 25m. The trawl was hauled for about 1h at the speed of 2–3 knots in each site, standardized to 1h haul with 2 knot vessel speed (i.e. CPUE of kn*h). The logarithmic transformed relative catch was used as the response variable to reduce data heterogeneity and to avoid the undue effect of outliers [38–40].

The predictive variables had two categories, environmental variables including sea surface salinity (SSS), sea surface temperature (SST), sea bottom salinity (SBS), sea bottom temperature (SBT), water depth and sediment type, and the spatio-temporal variables including geographical positions (i.e. longitude and latitude) and survey years. Temperature, salinity and depth were recorded using a CTD system (XR-420) in each sample site. The sediment types included sand, sandy silt, sand-silt-clay according to Shepard’s nomenclature of sediments [41] and were treated as factors in the analysis. Data were provided by the College of Environmental Science and Engineering, Ocean University of China (unpublished data). As there were substantial variations in species abundance among years due to the population dynamics rather than distribution patterns, survey years were included in the SDMs as a factor to adjust the fluctuation of relative abundance. Considering the differences in habitat and distributional pattern among seasons, and particularly the migration of *P*. *trituberculatus*, the spring and autumn data were treated separately in models and subsequent analyses (Table 1). That is, we assumed a relatively consistent distributional patterns within the same seasons and built the models for each season individually. The model for *P*. *trituberculatus* was omitted for spring due to its low prevalence as a result of seasonal migration.

**Table 1:** The description of data attributions used in the SDMs.

Environmental variable	Spring	Fall
year	2011, 2013, 2014, 2015, 2016	same as spring
sea surface salinity (SSS)	28.69–31.96	27.54–31.89
sea surface temperature (SST)	10.87°C-19.12°C	17.76°C-25.85°C
sea bottom salinity (SBS)	28.36–32.02	21.75–31.94
sea bottom temperature (SBT)	9.07°C-17.95°C	17.77°C-25.89°C
depth	6.37m-36.64m	3.77m-39.86m
sediment	sand, sandy silt, sand-silt-clay	same as spring
longitude	119.42°E-121.08°E	same as spring
latitude	34.42°N-35.58°N	same as spring

We used variation inflation factor (VIF) to examine the collinearity between predictive variables before model construction [42]. The VIF value of variable that was higher than 3 implied substantial correlation with other variables [42], thus were omitted.

### Statistical methods

GAM, RF and ANN were used to develop a set of species distribution models. Among these statistical methods, GAM was one of the most widely used methods in SDMs, whereas RF and ANN had many strengths over the traditional regression-based methods [24, 29, 43], such as efficient recognition of data patterns, independence of particular functional relationships, free-assumption of data properties, and the ability to accommodate interactions among variables without a priori specification [27].

#### Generalized additive model

Generalized additive model (GAM) is a non-parametric extension of generalized linear model (GLM) [44]:

$$\text{g}\left(Y\right)=\alpha +\overset{n}{\underset{i=1}{\sum }}f_{i}\left(x_{i}\right)$$ (1)

Where g() is the monotonic link function that establishes a relationship between the mean of the response variable and predictive variables, $f_{i}$ is a ‘smoothed’ function of explanatory variables, which enables to flexibly describe non-linear relationships [34, 45]. α is the intercept, and n is the number of explanatory variables.

#### Random forest

Random forest (RF) is an ensemble learning approach that generates multiple regression or decision trees [46, 47]. RF often shows satisfactory performance on prediction and gains increasing attention in a wide range of research areas. This method is typically implemented with the following steps [48]:

(i) Draw *n*_*tree*_ bootstrapped samples of the training dataset from the original data. (ii) Build multiple classification or regression trees with the bootstrap samples, in which each node of the unpruned tree is split by sampling *m*_*try*_ variables randomly and the best split is chosen automatically. (iii) Aggregate these units of tree information to attain the output.

In our study, the number of trees (*n*_*tree*_) was set to 2000, and we trained models with different *m*_*try*_ values and chose the optimal *m*_*try*_ = 2 when RF performed best.

#### Artificial neural network

Artificial neural network (ANN), inspired by the structure and activity of human brain, is a powerful tool for ecological issues that are difficult to be recognized or predicted by traditional statistical methods [27]. There exist many types of ANNs, but a common type and the one used in this study is specified as one hidden-layer with a feed-forward network trained by a back-propagation algorithm [49]. Specifically, the network is constituted by three layers of neurons: an input layer at which predictive variables are received, a hidden layer with complex connections, and an output layer with one or more neurons to make predictions. The number of neurons in the hidden layer is determined by minimizing the tradeoff between bias and variance [50]. Here, our study selected 5 hidden neurons in the network according to the performance of training models. The connection weights between neurons of different layers were adjusted to minimize the prediction error when training ANNs [25]. The models were implemented using the R packages *mgcv*, *randomForest*, and *nnet*, respectively.

### Model development and evaluation

Predictive variables were examined in the process of model development. The significant variables were selected using a stepwise variable selection procedure, which started with a null model and added one more predictive variable to the present model at each time step. For GAM, Akaike Information Criterion (AIC) [51] and Chi-square test among nested models [21] were used in variable selection for GAM, and the percentage of variance explained by the model (“variance explained”) was used for RF. The contribution of each variable to the final model was measured by the ‘percent deviation explained’ and the IncMSE value (i.e. the changes of mean square errors) in GAM and RF, respectively [46, 47]. For ANN, Garson’s algorithm [52] modified by Goh [53] was used to select predictive variables and determine their relative contributions [54]. In addition, variance explained (VE) was used as the common measure to compare the fitting capability among different models:

$$\text{VE}=\left(1-\frac{\text{Var}\left(\text{residual}\right)}{\text{Var}\left(\text{y}\right)}\right)\times 100\text{\%}$$ (2)

Where Var(residual) denoted the residual variance, and Var(y) denoted the variance of original data.

Sensitivity analysis was used to visualize the relationships between predictive variables and predictions, for which we changed each variable across its range while fixing the levels of other variables [54]. Since the relationships produced by ANN depended on the initial values and were not constant, we produced 100 response curves for each predictive variable in ANN to illustrate the variations, while other modelling methods produced one response curve for each predictive variable.

The cross-validation approach was used to evaluate the predictive performances of the models [55]. For each iteration (n = 100), the original dataset was randomly partitioned into 80% observations as training set for model building and 20% observations as test set for model validation [56, 57]. We used two metrics, the relative root-mean-square-error (RRE) and the coefficient of determination (R^2^), to evaluate the accuracy and precision of model prediction [27, 43, 58]. The degree of model overfitting was indicated by the difference of R^2^ between model fitting and model validation. The RRE measured the deviation of observed values and predictions, for which a smaller value implied improved predictability.

$$RRE=\frac{\sqrt{\frac{\sum_{i=1}^{n}{\left(O_{i}-P_{i}\right)}^{2}}{n}}}{O_{max}-O_{min}}\times 100\%$$ (3)

Here, *n* was the number of data points in the cross-validation, *O*_*i*_ was the observed values, *P*_*i*_ was the predicted values, *O*_*max*_ and *O*_*min*_ were the maximum and minimum values of observation.

Additionally, the standard errors of RRE and R^2^ were estimated as the measures of model stability, i.e., the robustness of predictability on random datasets [18, 29]. Multi-way ANOVA was used to identify the relative importance and interactions of three factors (i.e. modelling method, species, and season) on the variation of predictability [29].

### Distribution mapping

We used the Finite Volume Coastal Ocean Model (FVCOM) [59] to project the predictive variables over the whole area for mapping crab distributions. In this study, 64392 grid points were extracted from the FVCOM developed in Haizhou Bay (calibrated by College of Environmental Science and Engineering, Ocean University of China), including data of temperature and salinity by depth and time. The sediment types of these grid points were extracted from the same sediment map above. These environment data being estimated by the FVCOM were used to hindcast portunid crab distribution using the fitted models. The spatial and temporal variations of their potential distributions were shown and compared among the aforementioned modelling methods.

## Results

### Model fitting

VIF test suggested that SST showed multicollinearity with other variables. Thus, we used SBT in lieu of SST as a candidate predictive variable. Overall, sampling year, SBT, SBS, and sediment type were the most important factors affecting spatial distributions for different species and among survey seasons (Table 2). However, the fitted SDMs presented differences among species, modelling methods, and two seasons, regarding the retained predictive variables and their relative importance. ANNs included more variables in the fitted models than other approaches and showed much better explanatory performances, whereas GAMs included fewer variables and exhibited reduced performance for model fitting (Table 2).

**Table 2:** A Summary of fitted models for three crab species in spring and fall.

Species	Season	Model	Relative importance (%)	Variance explained (%)	The determination coefficient (R^2^)	$\Delta R^{2}$
*C*. *bimaculata*	Spring	GAM	depth(18.1)>year(7.1)>longitude(6.6)> SBT(2.4)	34.8	0.22	0.13
		RF	longitude(35.6)>year(24.3)>SBS(19.8)	67.3	0.28	0.5
		ANN	SBS(14.7)>SBT(11.5)>latitude>year(11.1)>sediment(7.7)	91.7	0.23	0.73
	Fall	GAM	year(12.2)>SBS(10.6)>SBT(3.6)	27.2	0.09	0.13
		RF	SBS(23.1)> sediment(19.0)>SBT(11.0)	58.7	0.11	0.61
		ANN	SBT(14.4)>year(13.4)>SBS(9.2)> sediment(6.1)>depth(6.0)	89.4	0.18	0.76
*C*. *japonica*	Spring	GAM	depth(20.9)>latitude(5.6) >year(5.3)> SSS(3.8)	36.2	0.18	0.25
		RF	Latitude(40.7)>SBT(31.4)>year(31.0)	70.5	0.20	0.59
		ANN	SBT(19.2)>depth(14.9)>year(11.2)> sediment(8.1)>latitude(4.6)	98.7	0.47	0.52
	Fall	GAM	SBS(11.8)>longitude(9.4)> SBT(1.0)	27.5	0.18	0.20
		RF	depth(36.9)>sediment(20.4)> SBS(12.8)	65.6	0.27	0.53
		ANN	SBT(16.3)>sediment(15.2)>latitude(14.5)>longitude(11.3)>year(6.4)	96.3	0.31	0.66
*P*. *trituberculatus*	Fall	GAM	year(35.7)>SBT(17.9)>sediment(1.0)	55.7	0.40	0.19
		RF	year(78.6)>SBT(22.4)	63.7	0.34	0.36
		ANN	year(14.9)>SBS(9.0)>SBT(8.1)> latitude(7.7)>sediment(7.3)	95.6	0.35	0.62

Note: Only the variables included in the optimal models were shown in the table. Relative importance referred to the contribution of predictive variables that retained in fitted models (GAM was based on ‘deviation explained’, RF was based on the percentage of IncMSE, ANN was based on Garson’s algorithm). Variance explained by each model represented goodness-of-fit of model. The determination coefficient (R^2^) represented predictive performance in the latter section of model predictability. $\Delta R^{2}$ referred to the difference of R^2^ between training models testing models.

**Fig 2:**
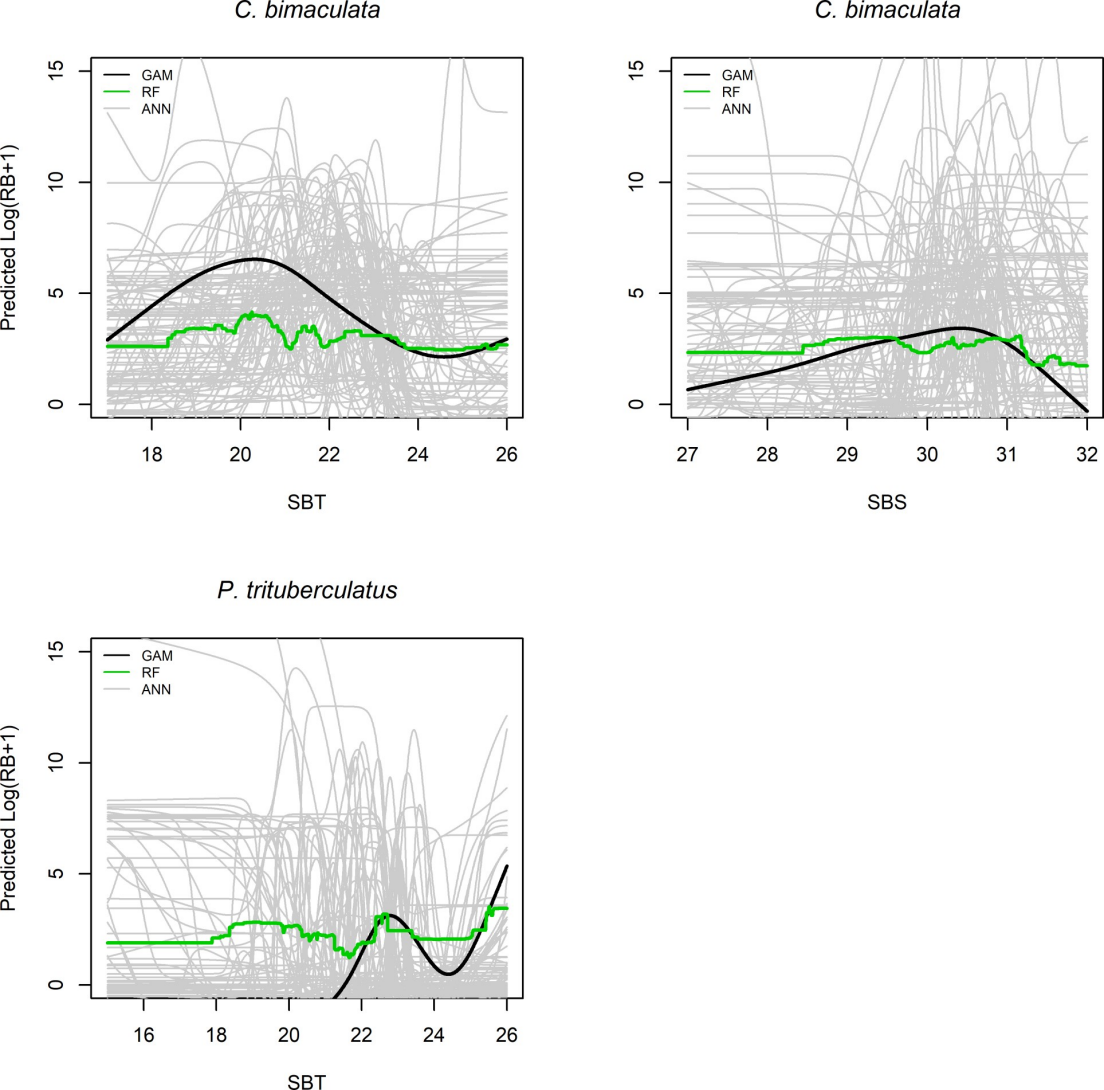
Contribution of influential environmental variables to the relative biomass (RB) of *C*. *bimaculata*, *P*. *trituberculatus* in fall. The results were derived from GAM, RF, and ANN, for which ANNs were examined with 100 repeats, and the other methods showed one curve.

We examined the sensitivity of predicted crabs’ biomass to the environmental variables selected for different models. The response curves exhibited the conditional effects of one variable on the predictions when the levels of other variables were fixed (Fig 2). GAMs showed simple relationships between predictive variables and predicted biomass, whereas ANNs and RFs reflected complex relationships. The response curves in ANNs changed substantially among 100 repeats, and the reflected effects of predictive variables varied among modeling approaches. In particular, SBT show different effects on predicted distributions among models.

### Model predictability

**Fig 3:**
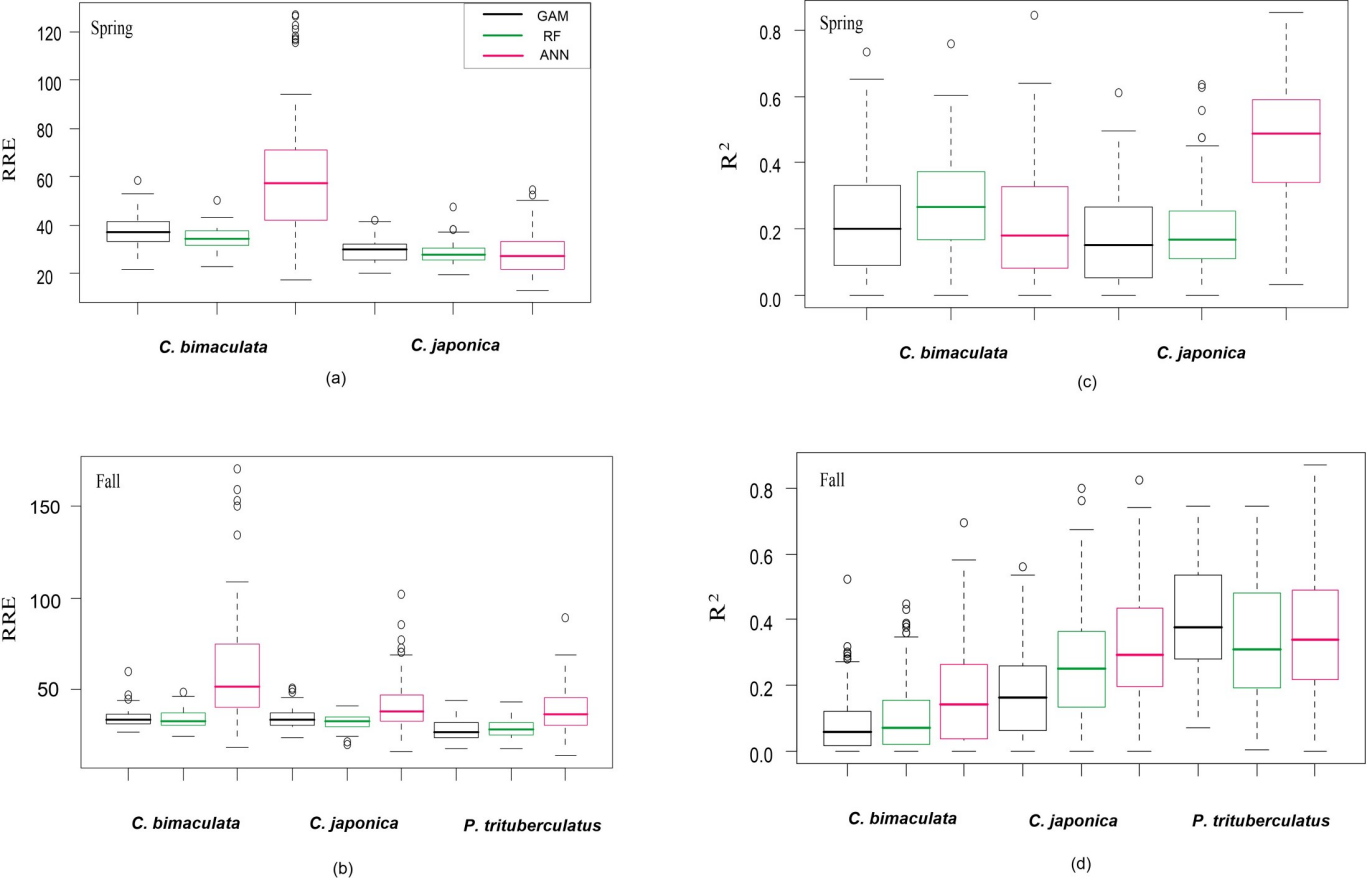
The predictability of crab distribution models measured by RRE and R^2^. Each plot showed one metric for all species during spring or fall, and *P*. *trituberculatus* was absent from spring sampling. The dispersals of RRE and R^2^ resulted from 100 times repeats in cross-validation.

For cross-validation, RRE of all fitted models ranged from 28 to 60 and R^2^ ranged from 0.08 to 0.47 (Fig 3). Among the three modelling approaches, no single method consistently outperformed others (Fig 3). GAMs and RFs provided better performances than ANNs on RRE, whereas ANNs exhibited higher R^2^ (representing predictive performance). The model predictability also varied among species, i.e., *P*. *trituberculatus* exhibited consistently better performances, and *C*. *japonica was* slightly better predicted than *C*. *bimaculata*. The same modelling method exhibited different predictability between seasons when modelling the same crab species, especially in R^2^ (Fig 3). Additionally, ANNs showed the largest difference between fitting capacity and predictability compared with other methods (Table 2).

Regarding model stability, the predictions of GAMs and RFs were more stable with lower standard errors in RRE and R^2^ (Table 3) comparing with ANN. The stability of GAMs and RFs were similar in spring but largely different in fall. Given the above, RF showed the best predictive performance with lower RRE and higher stability, followed by ANN with the highest R^2^.

**Table 3:** The stability of predictive capacity of crab distribution models measured by the standard errors of RRE and R^2^ in spring and fall.

Species	Model	Spring		Fall	
		SE of RRE	SE of R^2^	SE of RRE	SE of R^2^
*C*. *bimaculata*	GAM	0.06	0.15	0.05	0.08
	RF	0.04	0.15	0.05	0.10
	ANN	0.24	0.18	0.30	0.16
*C*. *japonica*	GAM	0.05	0.14	0.05	0.13
	RF	0.04	0.13	0.04	0.17
	ANN	0.08	0.19	0.14	0.19
*P*. *trituberculatus*	GAM			0.06	0.16
	RF			0.05	0.19
	ANN			0.12	0.20

Note: The standard errors of RRE and R^2^ were calculated by using 100 cross-validation results for each model.

ANOVA suggested all three factors were significant for predictability (Table 4). The modeling method showed a greater influence than species and season on RRE, whereas species was the most influential factor for R^2^. In addition, the interactions between method and both season and species were significant, that is, the modelling methods performed differently among seasons and species. The relatively weak effect of the method to season interaction suggested that the model predictability was relatively stable among seasons, whereas the large method to species interaction effect translated to unstable performances of modelling methods among species.

**Table 4:** The effect of influential factors on model predictive performance examined by ANOVA.

Criteria	Factors	SSE	Pr(>F)
RRE	method	5.730	< 0.001
	species	4.745	< 0.001
	season	0.317	< 0.001
	method:species	3.322	< 0.001
	method:season	0.223	< 0.001
R^2^	method	2.50	< 0.001
	species	6.84	< 0.001
	season	1.59	< 0.001
	method:species	2.97	< 0.001
	method:season	0.14	0.069

Note: SSE referred to the sum of square errors of REE or R^2^ attributed to each influential factor. (method: species) and (method: season) denoted the interactions between method and species or season, respectively.

### Mapping crab distributions

**Fig 4:**
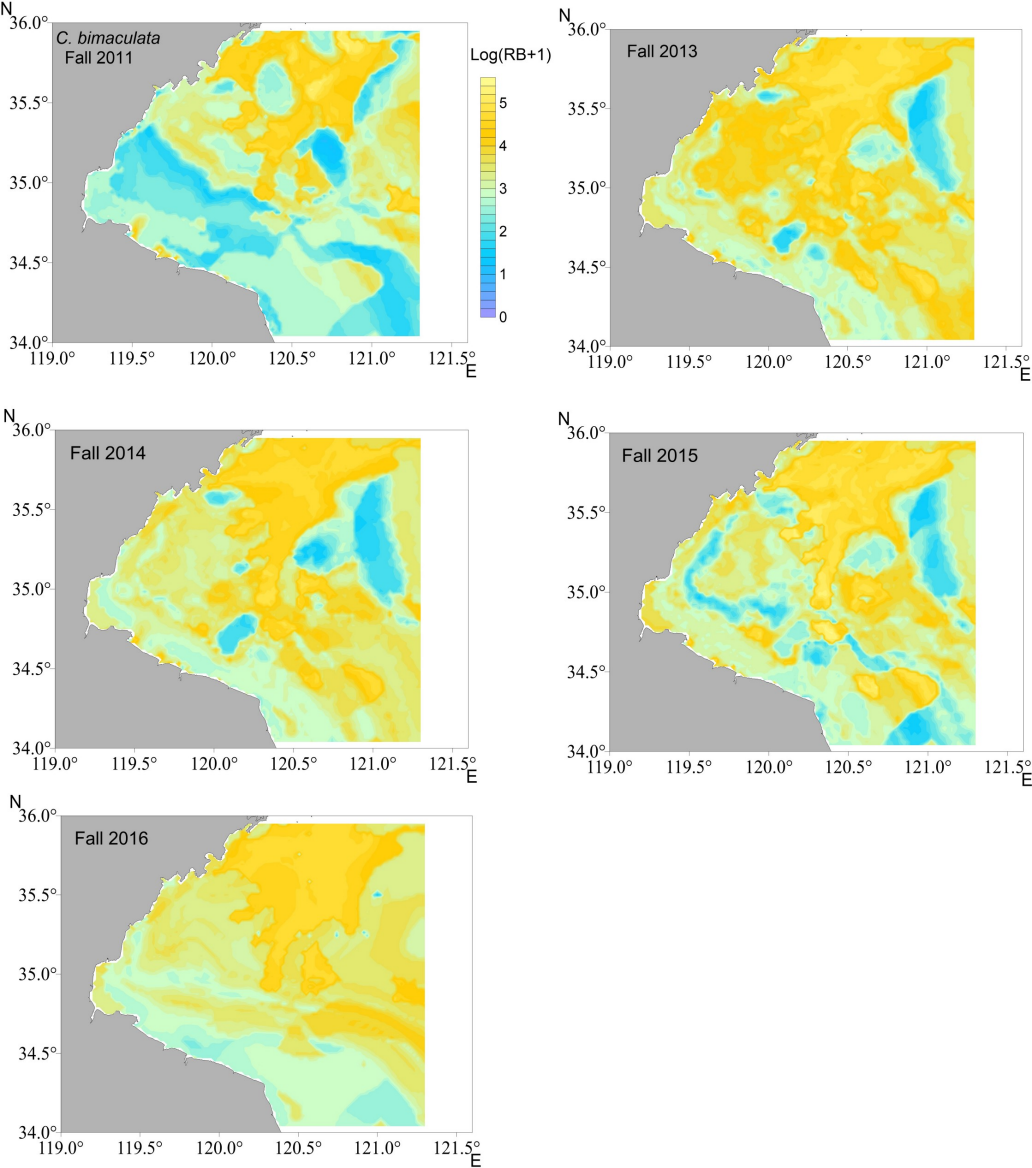
Spatial distribution of relative biomass (RB) for *C*. *bimaculata* in fall of each survey year predicted by random forest (RF) in Haizhou Bay.

**Fig 5:**
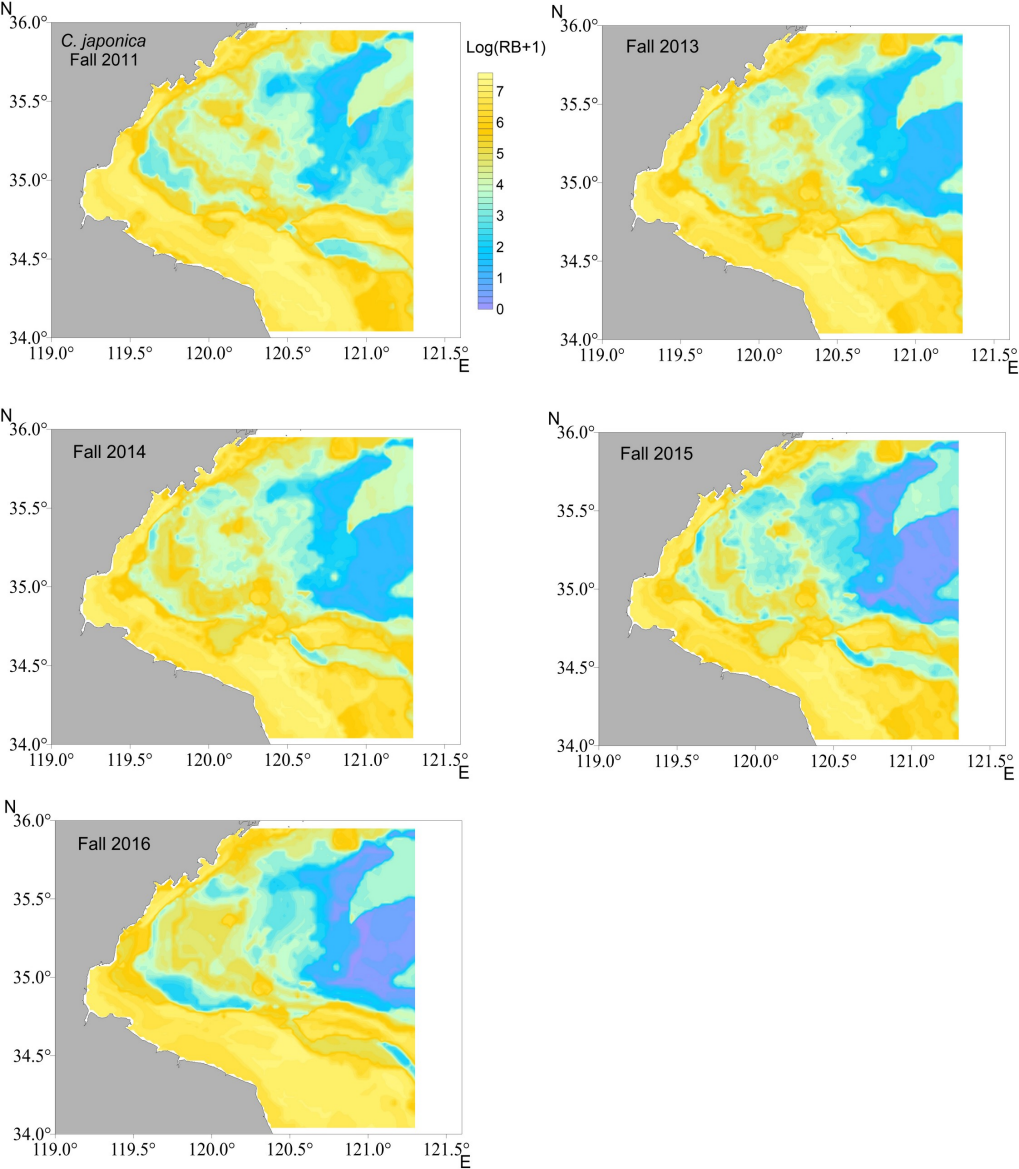
Spatial distribution of relative biomass (RB) for *C*. *japonica* in fall of each survey year predicted by random forest (RF) in Haizhou Bay.

**Fig 6:**
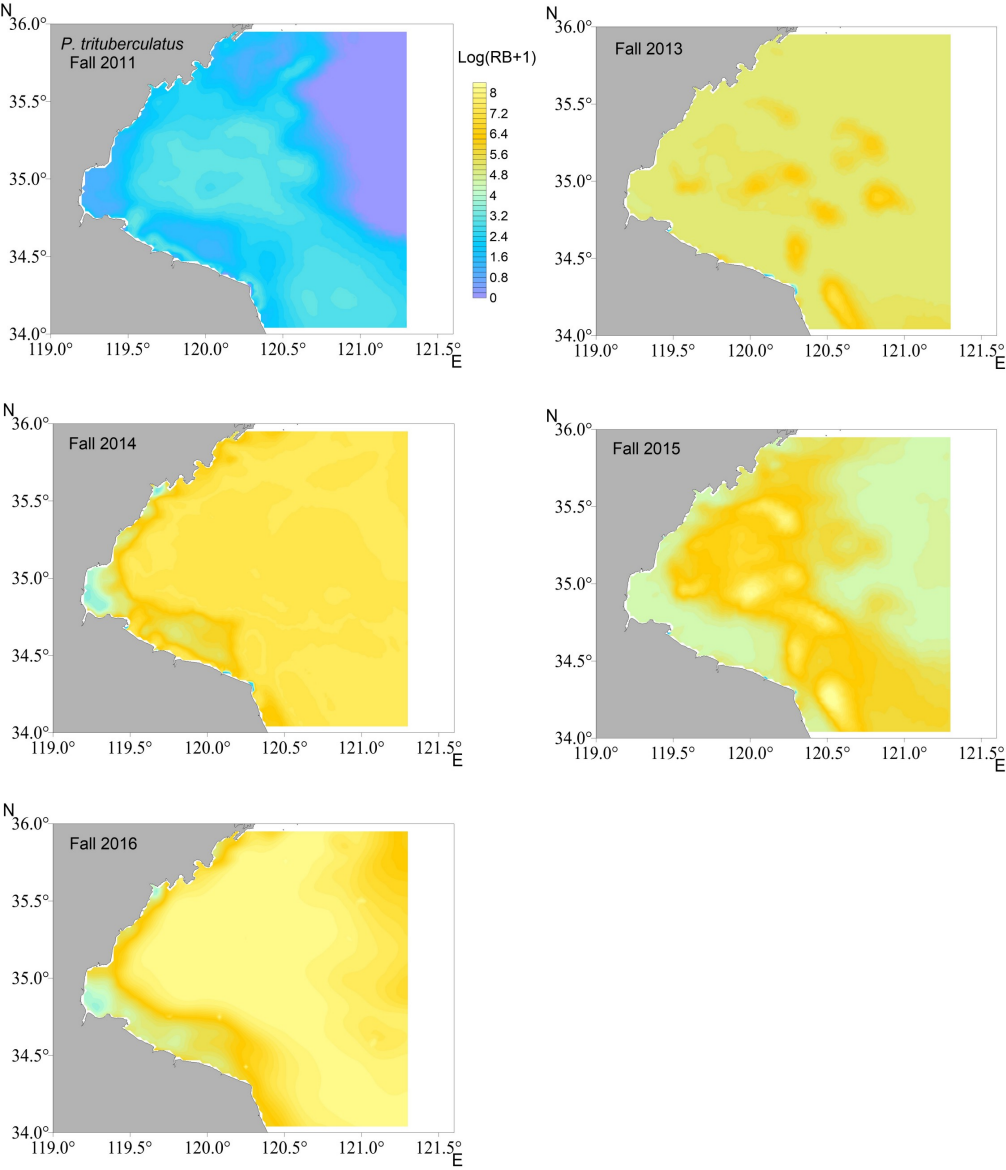
Spatial distribution of relative biomass (RB) for *P*. *trituberculatus* in fall of each survey year predicted by generalized additive model (GAM) in Haizhou Bay.

The distributions were mapped in each year using the most reliable models for each crab species i.e., RF for *C*. *bimaculata* and *C*. *japonica*, GAM for *P*. *trituberculatus*, respectively. The smaller biomass of *C*. *bimaculata* was predicted to be mostly in southwestern Haizhou Bay (Fig 4, results in fall as examples), whereas *C*. *japonica* was mainly located in the southwestern coastal waters (Fig 5). *P*. *trituberculatus* was predicted to distribute more evenly in the survey area (Fig 6). There was a substantial difference in the predicted density of *P*. *trituberculatus* between 2011 and the other years.

The predicted distribution maps were substantially different among modelling methods. Using *C*. *japonica* as an example, the results of GAM were similar to RF, showing higher biomass in the southwestern coastal waters in fall, whereas the results of ANN were substantially variable among survey years (S1 Fig). Considering seasonal differences in predictions, the maps showed that *C*. *japonica* tended to live nearshore, and the distributions were more stable in spring than that in fall (S2 Fig).

## Discussion

Identifying reliable models for projecting species distributions is important for fisheries conservation, management, and spatial planning [60]. The present study showed a comprehensive framework for model assessment in regards to fitting performances, species response curves, predictive capacity, and model stability. For the three species, no method consistently outperformed others. Our results highlighted the advantages and shortcomings of the models. In particular, we found RF was the most reliable method with robust predictions. In addition, the predictive performances were more variable among species than among modelling methods, which was consistent with previous studies [24, 33, 51, 61], suggesting that individual traits of a species should be highlighted in the choice of appropriate methods. Based on our results, we recommend use of multiple modelling approaches to generate more robust predictions for fisheries management [60].

In this study, all three approaches showed substantially better performances with training data compared to that with test data, implying a risk of overfitting. This may be attributed to both the complex species response to environmental variables and the limited data availability [62, 63]. Among the three modelling methods, RFs provided the best predictability and stable predictions over years but had a lower R^2^ compared to ANNs. Actually, the relative predictive capacity of ANNs and RFs varied greatly among studies with respect to different objectives and circumstances of their applications [35, 43, 64]. For instance, some studies suggested that RFs had advantages over ANNs in relation to avoiding overfitting [65] and simple adjustment to parameters [35], whereas ANNs could be adaptively trained to solve more complex ecological relationships [25, 27]. For modelling response curves, the simple patterns provided by GAM appeared to be more reasonable, whereas the complex relationships identified by ML methods did not necessarily mean they were unrealistic, because species-environment responses often tend to be complex, even after accounting for interactions between variables [21]. Given the synthetical evaluation of models, RFs showed better tradeoff among predictability and ecological interpretability and were more suitable for the crabs’ fisheries management.

Examining the species responses to environmental factors was conducive to understanding physiological and behavioral characteristics of different species [66]. SBT and SBS, the key environmental variables for the three crabs, have been shown to play a decisive role in many short living species [13]. *C*. *japonica* showed a low biomass at the temperature range 10–13°C, consistent with its preference of warm temperature. Likewise, SBS significantly influenced the distribution of *C*. *bimaculata*, indicating the preferred range of salinity 29–31. *P*. *trituberculatus* showed no optimum temperature range but instead exhibited more than one peak in the response curve (Fig 2). This result might be partly due to the ongoing southward migration of *P*. *trituberculatus* in the fall, which coincided with decreasing northern water temperatures. The latitude also showed large effects on crabs’ distributions which might be related with habitat differences in the north and south of the bay. Moreover, there was no guarantee that the determinant variables were included in our analysis, such as dissolved oxygen [17], precipitation and food availability [22].

Although the three crabs are closely related in taxon, their corresponding SDMs showed substantial variations in predictive performances, which were consistent with previous studies [61, 67]. It should be noted that different biological and life history traits may influence model capacity to capture species-environment relationships [16, 30, 68]. The large body size of *P*. *trituberculatus* may enable the species to hold on preferable environmental conditions when there are environmental fluctuations [68], resulting in better predictions. On the other hand, *C*. *bimaculata* is characterized by small size and high prevalence, which may result in some individuals staying in less satisfactory habitats, and therefore explain lower model prediction power [68]. In addition, different spawning activities and range sizes of three crabs may also influence the model performances through making their environmental requirements difficult to be describe [69].

Annual variation of abundance in fall and the absence in spring led to substantial uncertainty in the fishery management of *P*. *trituberculatus*. As the population of *P*. *trituberculatus* have dramatically declined over the last few decades [70], the risk of uncertainty should be explicitly and carefully accounted for in the future fishery management strategy. On the other hand, the annual populations of the other two species in fall were more robust when using their best fitted models therefore fishing effort might accommodate to this pattern for improving fishing efficiency. However, the distribution maps of *C*. *japonica* predicted by suboptimal ANN in fall showed variations among years, this result alerted managers to combine multiple models to inform the stock assessment.

Several conclusions of this study were highlighted for future SDM practices and the management of crab fisheries. For example, the performances of the modelling methods were relatively stable among seasons but varied substantially among species, implying that seasonality might be less concerned when choosing suitable modelling techniques for species. Besides, the high SE of REE and R^2^ suggested that the performances of ANN were not robust although they provided superior model fitting. The complex model structures implied that sufficiently large sample size of data should be desired in the use of ANN as well as other ML methods. In particular, *C*. *bimaculata* was characterized by wide tolerances in salinity and temperature [71] making it hard to be simulated. A larger sample size may benefit robust establishment of environmental requirements for this species. Long term climatic variations might be influential for species distributions, but were not incorporated due to the relatively short survey time series and improper resolutions [61]. Importantly, the SDMs in this study did not include consideration of biotic interaction and competitive exclusion [72, 73], which would be critical to correctly reflect realized ecological niches. These problems should also be considered in future studies.

## Supporting information

S1 Excel Relevant data used in model development. The unit of relative biomass is g/h.
(XLSX)

S1 Fig Spatial distribution maps of relative biomass (RB) for *C*. *japonica* in fall of 2011, 2013 and 2014 predicted by three modelling methods (i.e. GAM, RF and ANN) in Haizhou Bay. (EMF)

S2 Fig Spatial distribution maps of relative biomass (RB) for *C*. *japonica* in two seasons of 2011 and 2013–2016 predicted by artificial neural network (ANN) in Haizhou Bay. (EMF)

S1 Table Summary of fitted GAMs for three crab species in spring and fall. (DOCX)
