# Supplementary material for: Modelling the spatial distribution of three Portunidae crabs in Haizhou Bay, China
Source: PLoS One. 2018 Nov 14;13(11):e0207457. doi: 10.1371/journal.pone.0207457 (PMC6235385; doi:10.1371/journal.pone.0207457)
Supplement: S1 Table — (DOCX) [file pone.0207457.s004.docx]

| species | season | model | Evaluation  standard | Deviance  explained |
| --- | --- | --- | --- | --- |
| *C. bimaculata* | Spring | GAM | AIC=426.62 | 31.7% |
|  | Fall | GAM | AIC=422.65 | 22.3% |
| *C. Japonica* | Spring | GAM | AIC=450.11 | 41.2% |
|  | Fall | GAM | AIC=459.29 | 35.7% |
| *P. trituberculatus* | Fall | GAM | AIC=430.68 | 58.3% |
